# Comparison of Motor Learning Abilities Using Balance Training in Young and Senior Women

**DOI:** 10.33549/physiolres.935699

**Published:** 2026-04-01

**Authors:** Karla KOTKOVÁ, Yvona ANGEROVÁ

**Affiliations:** 1 Department of Rehabilitation Medicine, First Faculty of Medicine, Charles University and General University Hospital in Prague, Czech Republic

**Keywords:** Motor learning, Balance skills, Visual biofeedback, Aging

## Abstract

Motor skills learning is particularly relevant in rehabilitation settings, where patients often need to relearn movements that were lost due to injury or disease. The aim of the study was to assess the ability of motor learning in elder age, using balance training on a stabilometric platform (Homebalance) with visual biofeedback. Both the experimental group (senior women) and the control group (young adult women) underwent the same balance training program, after which demonstrated improvements in velocity and accuracy of movements changing position of center of gravity (COG). The groups differed in the absolute values of the times achieved, but the improvement during training showed similar dynamics. The initial improvement phase lasts approximately the same amount of time for young and old participants. The next training period (from the 6^th^ week) shows a slower (still statistically significant) improvement dynamic in the young adult women group and a minimum improvement in the senior women group. All participants in both groups exhibited greater balance control following the intervention - both groups were able to apply their improved skills even in a non-trainable sequence with random target appearance. The results demonstrate that motor learning of balance skills using visual biofeedback is possible even in the elderly population, and that the dynamics of the process is similar in young and older people.

## Introduction

Motor skills learning is a complex process that involves the acquisition, developing and refinement of movements through practice and experience. This process is crucial for performing everyday activities and specialized tasks, and it is influenced by various factors (cognitive, motor, and environmental).

Motor learning theory according to Fitts and Posner just in 1967 suggested three main phases of motor learning. The first phase they called cognitive. It needs high degree of cognitive activity to understand the task, to develop and try various strategies to realize the task and to choose the best one). The second phase is called associative stage. The person begins to refine the skill, which means to improve their movements through practice, reducing errors and increasing efficiency. It can take days, weeks or months depending on the exerciser and the intensity of the workout. The third stage of skills acquisition is called the autonomous stage. The person can begin to give attention to other aspects of the skill or focus on a secondary task, because the acquired skill now needs minimal cognitive effort [1].

Early stages of learning engage cognitive processes like working memory and error detection, which are associated with the prefrontal cortex and striatal regions. As learning progresses, these skills become more automated, reducing the need for cognitive control and shifting reliance to motor cortical regions [2]. The cognitive phase can be characterized as a period of inconsistent performance and the greatest improvement/progress, but with many errors leading to the selection of an appropriate strategy. The association phase is characterized by smaller advances, persistent inconsistent performance, fewer errors. The autonomous phase is characterized by already automated performance with a low error rate even in a potentially disruptive environment [3].

One of the manifestations of neuroplasticity is the ability of neural circuits to restore their function that has been disrupted by structural impairment. Plastic changes in neuronal systems are applied in both physiological and pathological situations and use general mechanisms of cellular functions [4,5].

In clinical rehabilitation practice, we attempt to influence the impaired function with a large and diverse range of information directed to the damaged area, so that the best possible reparative or additional mechanisms can be formed, i.e. by using different therapeutic procedures of physiotherapy, occupational therapy, speech therapy and neuropsychology. Based on an individual examination of each patient, we need to find the optimal therapeutic approach [6,7].

That is why we are interested in recognising the mechanisms of motor learning, its course, dependence on age or type of training.

Feedback helps to correct errors and refine movements, which is why some form of feedback is essential for motor learning. It includes all the sensory information that is available as the result of a movement that a person has produced. Intrinsic feedback means information from the various sensory systems (e.g. visual information recording the accuracy of a movement, somatosensory information about position of body segments….). Extrinsic (or augmented) feedback supplements intrinsic feedback – extends and specifies information during or at the end of the task [1].

For the purpose of augmented feedback, the biofeedback procedures can be used. Basmajian’s classic definition defines biofeedback as a technique that uses electronic or other devices to monitor and amplify body functions, enabling individuals to become aware of and consciously control physiological processes that are normally considered involuntary [8]. Those procedures are based on specific devices that assist the body functions by displaying a selected task. The principle is the continuous recording of the function by the device and displaying it for monitoring by sight and/or hearing. Visual biofeedback is enabled by devices that combine the recording of one (or more) body functions and their continuous visual display (e.g. on a tablet screen) [9]. In our case visual biofeedback provides information about the direction and magnitude of the change in the position of the center of gravity (COG) and thus enables smooth correction of the trained skill. Consistent training is necessary for reinforcing the neural pathways and improving skill performance [10,11].

A stabilometric platform can be used for balance training skill development. Stabilometric platforms record the trajectory of the COG (a point around which the force of gravity appears to act), and project it onto a two-dimensional support base. The center of mass (COM) is defined as the hypothetical point at which the weight of the entire body is concentrated, determined as the weighted average COM of all body segments. Although gravity acts on all body segments, its final effect on the body is realized through COM [1]. The location of the COM is not fixed but depends on body position [12]. The projection of the body’s COM onto the base of support plane is called COG. In a static position, the COG must always be located within the base of support [13]. The degree of stability depends on four factors: the height of the COM above the support base, the size of the support base, the location of COG in the support base, and the weight of the body. Stability increases with a lower COM, a wider base, a projection of the trajectory as close to the center of the support base as possible, and possibly an increase in the weight of the object. Any deviation from the equilibrium position causes a shift in the COM and COG [14]. The base of support is understood as the imaginary surface shaped by the outlines of the person’s feet.

Balance can be defined as the ability to maintain the COM above the base of support [15]. The balance of the human body is a complex process that requires the connection of incoming afferent sensorimotor information to the brain and the execution of an appropriate response by the musculoskeletal system. In a healthy person, the body position is ensured reflexively. Body balance is primarily controlled by the motor centers of the brainstem, especially the reticular formation and vestibular nuclei, through the coordination of positional, postural and righting reflexes. Afferentation comes mainly from proprioceptors and from the statokinetic sensor, the most important efferent pathways are the vestibulospinal and reticulospinal pathways [16].

An important factor for the balance maintenance is the ability to respond to changes in the position of COM and COG - the speed of change in the position of them is a key skill for keeping balance in posturally complex situations (slipping, tripping, falling, carrying a load, distraction - dual task) [17]. The speed of reaction is affected by structural damage to the above-mentioned mechanisms, but also by age - which, for example, endangers seniors in unexpected and unpredictable situations [18]. The main goal of our study was to realize, whether and how the ability to voluntarily change the movement of the COG can be influenced by training and what is the difference between young and older women.

## Methods

### Participants

For study feasibility, we selected groups with high compliance, i.e., women who are regularly engaged in non-specific physical activity. Two groups of women (“senior” 65–75 and “young adults” 18–24 years) participated in this experiment. Thirty-one younger adults (M = 20,967, SD = 0,967) and 30 senior (M = 67,567, SD = 2,996). Young adults were recruited from the students of physiotherapy of The First Faculty of Medicine, Charles University in Prague. The senior women were recruited from persons attending senior fitness exercises at a Reconditioning Center in Prague 2, and those attending of a course of Greek dances in Prague 2. All recruited young adult volunteers completed the training program, in the senior group one participant did not complete the training for health reasons.

Inclusion criteria were age, gender, any experience with balance training. Excluding factors were balance disorders, severe structural changes of musculoskeletal system and/or neurological diseases with motor and/or sensory symptoms. All the seniors were very high functioning adults living independently, in good self-reported health and had no significant medical or neurological problems. All participants had normal or corrected to-normal vision. The study was conducted in accordance with the standards of the Ethics Committee and the Helsinki Declaration of 1975, revised in 2013. Approval for the research was granted by the Ethics Committee of the General University Hospital in Prague on October 16, 2014, reference number 161/14. Participants signed an informed consent form and were informed about the course of the study and the possibility of withdrawing from the study at any time.

## Materials

The Homebalance system, consisting of a stabilometric platform with a tablet, was used. The system was developed by the Spin-off Application Centre at the First Faculty of Medicine, Charles University in Prague in cooperation with the Faculty of Biomedical Engineering of the Czech Technical University in Prague. We used an earlier version of the device, where the tablet (screen size 21,5x 13,5cm) was placed in front of a standing person at a height allowing a direct view. The system has pre-set training sequences with defined different levels of difficulty. Individual training sequences were defined with the aim to gradually increase the difficulty in terms of the number of targets and their distance from the projection of the COG while standing with feet hip-width apart. Visual feedback was provided by a tablet presenting the task in the form of a sequence of gradually appearing targets. The tablet communicated with the platform using Bluetooth technology [19,20].

### Test

1. Side to side: stand hip-width apart, targets alternate side to side.
2. X shape, small: stand hip-width apart, targets alternate in diagonal pattern, movements return to the basic position after each excursion.
3. X shape, large: stand hip-width apart, targets alternate in diagonal pattern, movements return to the basic position after each excursion. Excursions are twice larger than in X shape, small test.
4. Spiral right: stand hip-width apart, targets follow
5. a pattern out from the center into a right-handed spiral with increasing distances.
6. Spiral left: stand hip-width apart, targets follow
7. a pattern out from the center into a left-handed spiral with increasing distances.
8. Random pattern: stand hip-width apart, the target sequence is randomly generated by the device.

Tests 1 to 5 were performed twice, the test 6 only once.

### Procedure

Both groups trained with an identical schedule: once a week for ten consecutive weeks, on a stabilometric platform with visual feedback, i.e. they could watch the movement of their COG projection in the training field on the screen. Each sequence was performed twice in a quick succession, with the exception of the “random” sequence – performed once.

The speed and accuracy of movement was practiced by deflecting the body towards individual targets of the training sequence. The sequences were predefined having increasing difficulty (number of points, distance of points, directions of distribution of points) and led the subjects to increasingly more complex body movements. There were 11 sequences in total, ten of which had a fixed distribution of target points. The eleventh was randomly generated by the computer.

### Data analysis

The device recorded the time of the given sequences performed. These data were first presented graphically as time series (ten values measured at weekly intervals) for comparison of the results of young and senior subjects. Time series were separately compiled for each sequence for absolute values, relative values (expressed as % of the initial value) and for improvement values from the previous measurement (i.e. for the last week). The data were considered to be approximately normal (given the range of samples, this assumption has a marginal impact) and the following characteristics were calculated: mean value, variance/standard deviation, two-sided 95 % confidence interval (CI) and t-test of the hypothesis that there was no improvement during the given period, against the hypothesis that there was improvement (i.e. one-sided hypothesis). The following periods were considered for this test: 1^st^ to 10^th^ week, 1^st^ to 6^th^ week, 6^th^ to 10^th^ week.[Table t1-pr75_365]

## Results

A total of 61subjects participated in the study in two groups: young adults (31) and senior women (30). Both groups differed in the speed of performance of the given sequence, group of young adult was able to finish the sequence more quickly, the initial velocity of young adults was better. The difference in the initial values of the two groups is large, so a direct comparison of the two groups cannot be used in statistical processing. Therefore, when comparing relative improvement (as a percentage of the baseline value), both groups showed statistically significant improvement in results comparing the difference between the first and tenth weeks. That means that young and senior probands are able to learn and that the group of senior women maintain the motor learning skills.

**Fig. 1:**
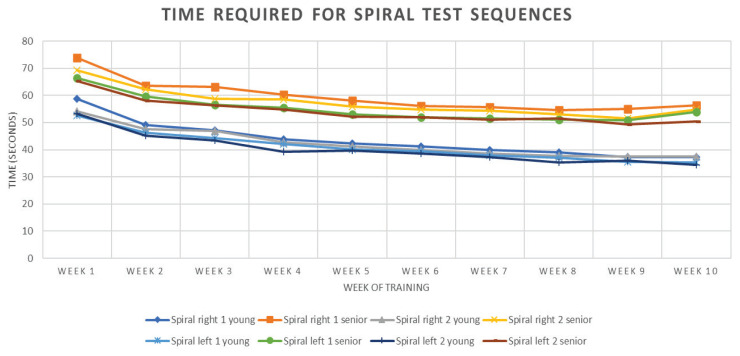
Time required for spiral test sequences – mean values.

The initial values are obviously different for senior and young adult group, but the dynamics of motor learning are comparable, with the difference in absolute values that was present at the beginning (see Fig. 1 showing “spiral tests” as an example). This suggests the mechanism, that condition learning is similar in both young and older age. Although the improvement is not statistically significant after the sixth week, the trend of practical improvement is there and should be the subject of further study.

The results confirmed the improvement of both groups in the initial phases of motor learning. The greatest improvement was achieved by both groups in the second week, with a progress until the 6^th^ week. The improvement of both groups was slower from the 6^th^ week onwards, still statistically significant in the group of young women, and statistically significant in the group of seniors only in the tests “spiral right 2” and “spiral left 2”.

The results show an improvement in both groups, but they varied depending on the type of sequence. Senior women improved mainly in sequences requiring a larger range of motion (“spiral right”), less in sequences requiring small ranges of motion and rapid change of direction of movement (“X shape small”). Young adults achieved more consistent improvements across all types of training, but there was also a greater improvement in the sequences with a greater COG deviation (the maximum improvement in “spiral right 1”).

The “random” test, where mental learning of the data sequence was impossible, demonstrated the ability to transfer motor skills to random reactions, i.e. to various common daily activities - again in both groups. In this test, an improvement was also evident in both groups, although the younger individuals did better and the variability of their results was smaller.

## Discussion

The aim of the study was to assess the ability of motor learning in elder age, using balance training on a stabilometric platform (Homebalance) with visual biofeedback. Both the experimental group (senior women) and the control group (young adult women) demonstrated improvements in velocity and accuracy of movements changing position of COG in each training sequence.

Balance is associated with ambulation abilities and quality of life. Moreover, balance is found among the factors potentially modifiable by physical activity [21]. Hence, balance training is an important strategy for prevention of hypomobility and falls not only in elder people, and knowledge of learning process of motor balance skills is important for planning training for older people and people with disabilities, e.g. after a stroke [22]. Several factors influence the efficiency of motor skills learning – age, motivation, attention, feedback, practice and repetition [2]. We were interested in the role of age and feedback.

The learning mechanisms that we are trying to use are closely linked to the capacities of procedural (non-declarative) memory in the restoration of movement. Non-declarative memory is phylogenetically old, it is usually located in primary and association cortical areas, in the cerebellum, thalamus and basal ganglia. It is a part of various behavioral manifestations. Memory traces are gradually created by multiple repetitions; they are manifested by improved performance in a certain activity and are difficult to express in words. For rehabilitation, the key role is played by the motor part of non-declarative memory (the formation of movement patterns, their temporal and spatial sequences) and the sensory part (the formation of perceptual schemes, e.g. storing patterns of procedures for processing certain sensory perceptions, professional skills, etc.). [16]. Age related changes in motor and cognitive functions can affect learning. While older adult may experience declines in motor performance, they can still achieve significant improvement with practice [23–27]. Although the performance of motor skills can be greatly affected by age, skill acquisition is relatively unaffected by age [10]. Study of Rajeshkumar [28] shows, that both young and older adult group adapted their movement equally well, older adult adapted as quickly as younger in sequence condition; however, in random condition the learning was slower.

The technology with visual biofeedback allows an increase in training intensity by interaction with computer, training in the area of execution strategies and visualization of functions, which would otherwise be difficult to imagine (movement of the COG). Visual biofeedback thus offers positive reinforcement, facilitating improvement in the execution of the task [29]. The other advantage of these techniques is that the patient watches his/her own current results, monitors the gradual improvement (in our case shortening of times necessary to perform the training sequences) and is thus motivated to continue in training. There are studies and reviews [11,20,22,30–32] that used virtual reality with visual biofeedback during physical therapy, and they confirm the ability of persons with disabilities or with elder age to exercise on stabilometric platforms.

The groups differed in the absolute values of the times achieved, but the improvement during training showed similar dynamics. The initial improvement phase, which perhaps corresponds to the cognitive phase, lasts approximately the same amount of time for young and old participants. The next training period (from the 6^th^ week), which probably corresponds to the associative phase, shows a slower (still statistically significant) improvement dynamic in the young adult women group and a smaller improvement in the senior women group.

All participants in both groups exhibited greater balance control following the intervention - both groups were able to apply their improved skills even in a non-trainable sequence with random target appearance. That demonstrates ability of motor learning and even application of learned skills also in elder age.

There are studies describing motor learning in older and younger individuals [26,33–35], but they used motion sequences focused on the upper limbs. However, similar results are described in Trewartha’s study [26]. Šajtárová and Janatová [32] used the same technical system in their studies and used motion sequences focused on movement related to body balance. They aimed at seniors with balance disorders and confirmed the positive effect of exercises with visual feedback [32,36].

The present study demonstrates that such training is viable, safe and effective in enhancing balance control skills in the standing position. The demographic data suggest the increasing number of seniors and the result of the present study demonstrate ability to undergo balance skills training in elder age and ability to improve balance skills and apply motor learning. The study also showed that new technologies, which older generations were unfamiliar with, can be used to train their motor functions. Seniors results demonstrated that they had previously reached the limit (peak) of their capacities. In clinical application, this probably means that shorter training sequences are sufficient and that repeating the entire training session is likely to be beneficial. This should be the subject of further research. For senior women, training sequences with a greater range of COG movement appear the most promising, and potential application in routine rehabilitation could be also the subject of further study.

## Limitations

Some limitations of present study should be pointed out. The study was carried out with sample composed of participants with high compliance – women who are regularly engaged in non-specific physical activity. Further studies should be conducted on larger sample based on gender-balanced group moderately physically active.

## Conclusions

This study confirms that motor learning of balance abilities using visual biofeedback is possible in elder population and the dynamics of process is similar in young and senior age. Motor skills learning is particularly relevant in rehabilitation settings, where patients often need to relearn movements that were lost due to injury or disease. Techniques using special task training with visual biofeedback can enhance motor learning and recovery. By understanding the principles of motor skills learning in age related consequences, rehabilitation practitioner and specialists can develop more effective training and rehabilitation programs tailored to individual needs. The more different technologies we can use, the greater our ability to select the most suitable training method for a specific patient, utilizing the relevant brain capacity. On the other hand, training must have very clear rules to avoid oversaturation and subsequent complications.

## Figures and Tables

**Table 1 t1-pr75_365:** Results of all test types – mean values of time (seconds)

test type	week 1	week 2	week 3	week 4	week 5	week 6	week 7	week 8	week 9	week 10
*side to side 1 young*	56.71	47.87	43.48	41.77	39.68	38.16	37.29	36.71	37.10	35.84
*side to side 1 senior*	63.09	54.21	51.79	50.85	51.06	49.24	49.30	49.12	48.27	47.50
*side to side 2 young*	52.94	45.06	41.52	40.71	38.81	38.10	37.06	35.77	35.32	35.65
*side to side 2 senior*	60.00	53.94	50.73	50.79	49.39	47.45	46.70	46.33	46.73	47.78
*X shape small 1 young*	36.48	34.97	32.10	31.10	29.90	29.19	28.74	28.16	27.26	26.26
*X shape small 1 senior*	43.97	40.09	39.12	39.00	37.76	37.39	37.06	36.67	35.82	38.06
*X shape small 2 young*	35.42	32.35	31.39	29.45	28.94	28.45	27.55	28.10	26.06	25.06
*X shape small 2 senior*	41.79	38.45	38.45	37.73	37.27	37.15	36.39	35.52	36.06	36.06
*X shape large 1 young*	52.10	46.26	40.58	38.77	37.23	36.26	35.10	34.65	34.48	33.26
*X shape large 1 senior*	60.58	55.91	52.82	51.73	49.15	48.61	47.73	47.03	47.39	48.56
*X shape large 2 young*	51.48	44.19	41.52	38.45	36.94	36.19	34.90	34.52	34.10	32.45
*X shape large 2 senior*	59.61	53.85	52.70	50.06	49.52	47.70	47.12	47.73	45.73	48.06
*spiral right 1 young*	58.84	49.10	47.06	43.87	42.39	41.23	40.00	39.13	37.23	37.29
*spiral right 1 senior*	73.91	63.55	63.12	60.33	58.09	56.18	55.76	54.61	55.00	56.39
*spiral right 2 young*	54.06	47.52	47.00	42.71	41.13	39.81	38.65	37.81	37.42	37.39
*spiral right 2 senior*	69.36	62.24	58.67	58.55	55.94	54.79	54.27	53.09	51.55	54.83
*spiral left 1 young*	52.71	46.26	44.29	42.03	40.06	39.55	38.00	37.13	35.52	35.26
*spiral left 1 senior*	66.33	59.64	56.45	55.39	52.94	51.97	51.52	51.18	50.76	54.00
*spiral left 2 young*	53.32	45.19	43.45	39.26	39.61	38.48	37.32	35.29	35.87	34.52
*spiral left 2 senior*	65.39	58.00	56.33	54.79	52.24	51.97	51.06	51.45	49.24	50.39
*random young*	57.94	52.45	51.45	48.94	48.10	47.42	46.55	45.23	44.74	43.39
*random senior*	68.48	64.33	60.70	60.79	60.85	58.67	57.70	57.61	56.79	57.78

**Table 2:** Dynamic of improvement in percentage of initial values for all tests.

test	week interval	young adult		senior	
		mean	sd	mean	sd
*side to side 1*	1→10	20.871^*^	9.133	17.278^*^	8.592
	1→6	18.548^*^	8.987	15.667^*^	8.265
	6→10	2.323^*^	3.779	1.611	4.738

*side to side 2*	1→10	17.29^*^	10.113	13.5^*^	10.469
	1→6	14.839^*^	10.589	13.556^*^	10.308
	6→10	2.452^*^	4.758	−0.056	4.384

*X shape small 1*	1→10	10.226^*^	6.1	7^*^	8.866
	1→6	7.29^*^	6.049	6.833^*^	7.958
	6→10	2.935^*^	3.047	0.167	3.353

*X shape small 2*	1→10	10.355^*^	6.115	6.278^*^	5.616
	1→6	6.968^*^	6.742	5.222^*^	6.732
	6→10	3.387^*^	3.678	1.056	3.343

*X shape large 1*	1→10	18.839^*^	7.984	13^*^	8.458
	1→6	15.839^*^	9.218	12.333^*^	7.043
	6→10	3^*^	3.835	0.667	4.362

*X shape large 2*	1→10	19.032^*^	7.037	14.5^*^	9.073
	1→6	15.29^*^	6.994	13.889^*^	10.454
	6→10	3.742^*^	3.51	0.611	6.241

*spiral right 1*	1→10	21.548^*^	10.728	22^*^	12.584
	1→6	17.613^*^	10.724	19.833^*^	12.493
	6→10	3.935^*^	4.355	2.167	7.814

*spiral right 2*	1→10	16.677^*^	9.891	19.556^*^	10.064
	1→6	14.258^*^	9.948	16.444^*^	9.319
	6→10	2.419^*^	4.456	3.111^*^	4.017

*spiral left 1*	1→10	17.452^*^	6.923	13.778^*^	8.119
	1→6	13.161^*^	8.621	13.778^*^	9.247
	6→10	4.29^*^	3.937	0	3.973

*spiral left 2*	1→10	18.806^*^	6.813	16.389^*^	8.859
	1→6	14.839^*^	7.531	11.889^*^	10.094
	6→10	3.968^*^	4.461	4.5^*^	6.297

*random*	1→10	14.548^*^	7.215	12.944^*^	6.92
	1→6	10.516^*^	6.951	11.222^*^	8.59
	6→10	4.032^*^	3.737	1.722	7.032
